# Structure design and implementation of a water cooling sub-20 nm multilayer Kirkpatrick–Baez mirror system in the Structural Dynamics beamline ID23 at HEPS

**DOI:** 10.1107/S1600577526007393

**Published:** 2026-08-05

**Authors:** Ruiying Liao, Haihan Yu, Jianye Wang, Xue Wang, Changrui Zhang, Wenkai Kang, Ling Fan, Chunxia Yao, Penghao Hou, Darui Sun, Fang Liu, Lu Zhang, Zhaoqiang Cui, Zhongrui Ren, Zina Ou, Ruzhen Xu, Duanyang Fu, Yuhang Wang, Pengcheng Li, Chenglong Zhang, Lingzhu Bian, Miao Zhang, Zhanxin Gu, Zhe Li, Zhibang Shen, Lidan Gao, Bingbing Zhang, Shanzhi Tang, Ming Li, Weifan Sheng

**Affiliations:** a Multidisciplinary Research Center Institute of High Energy Physics of the Chinese Academy of Sciences Beijing People’s Republic of China; b College of Nuclear Science and Technology University of Chinese Academy of Sciences Beijing People’s Republic of China; Bhabha Atomic Research Centre, India

**Keywords:** MLKB mirror system, stability structures design, cooling system, focal spot, eGaIn gap-filling, copper-braid cooling

## Abstract

The first water-cooling nano multilayer Kirkpatrick–Baez mirror system in the Structural Dynamics beamline (ID23) at High Energy Photon Source (HEPS) has been implemented. An Invar gantry is engineered to achieve a balance between light weight and stability. The cooling system, including eutectic gallium–indium (eGaIn), copper braids and a cooling water circuit with a multi-bend copper pipe in a compact space, decouples movement and mitigates vibration. The mirror cooling holders introduce less than 0.5 nm RMS height error and 0.1 µrad RMS slope error in the mirror surface shapes. A series of stability tests is applied to verify the mechanism structure. In a 1 h test assessment using a water-cooling flow of 4.5 L min^−1^, the system demonstrated a positional stability of 5.96 nm and an angular stability of 86.76 nrad from 1 Hz to 500 Hz. The focal spot size of 13.39 nm × 15.15 nm (H × V) at a photon energy of 21.8 keV demonstrates the system’s performance.

## Abstract 2

A water-cooling nano multilayer Kirkpatrick–Baez mirrors system has been implemented at ID23 HEPS. The focal spot size of 13.39 nm × 15.15 nm (H × V) at a photon energy of 21.8 keV demonstrates the system’s performance.

## 1. Introduction

The Structure Dynamics beamline (ID23) at the High Energy Photon Source (HEPS), China, aims at imaging and diffraction technology with multi-time resolution (420 ps to 20 µs) and multi-space-scale resolution (60 nm to 10 mm), facilitating *in situ* real-time diagnosis of dynamic and irreversible processes in dynamic compression science and additive manufacturing (Jiao *et al.*, 2018 ▸). To achieve extensive time and space resolution, ID23 primarily operates three experimental modes working in three distinct hutches: white beam mode in Hutch L, micrometre mode in Hutch M, and nanometre mode in Hutch N. For high temporal resolution in dynamic experiments, a substantial photon flux (10^16^ photons s^−1^) to the sample during a brief exposure necessitates a pair of short-period cryogenic permanent magnet undulators as a pink light source. The nanometre mode of ID23 is characterized by its integration of nanometre-scale spatial resolution with microsecond-scale temporal resolution to investigate the dynamic behaviour of materials. To address the thermal effects from the pink source on the mirror surface and to cool focusing optics elements, a multilayer Kirkpatrick–Baez (MLKB) mirror system is applied.

Kirkpatrick–Baez (KB) mirrors typically include two orthogonal grazing incidence mirrors. Theoretically, the focusing spot size may approach the diffraction limit (Liu *et al.*, 2011 ▸; Matsuyama *et al.*, 2006 ▸) and the mirrors are extensively used in synchrotron sources (Liu *et al.*, 2005 ▸; Barrett *et al.*, 2011 ▸; Kearney *et al.*, 2021 ▸; Gong *et al.*, 2016 ▸). Use of a multilayer coating would enhance the numerical aperture constrained by the critical angle, hence improving the focusing performance (Wang & Li, 2021 ▸; Chapman & Bajt, 2021 ▸; Underwood & Barbee, 1981 ▸). The size of the focal spot is a crucial index that influences the performance of the KB mirror system, including the stability of the mechanism, the quality of the polished surface, installation and the cooling scheme. At the Nano Imaging beamline (ID16A) and Nano Analysis beamline (ID16B) at the European Synchrotron Radiation Facility (ESRF), two nanofocusing MLKB mirror systems implement diameters of sub-30 nm and sub-50 nm, respectively (Barrett *et al.*, 2016 ▸). The hard X-ray nanoprobe beamline at Shanghai Synchrotron Radiation Facility (SSRF) introduces an MLKB focusing system together with its phase compensator system, enabling a two-dimensional focal spot of 26 nm ×17 nm (Jiang *et al.*, 2024 ▸). A system including a deformable mirror and a laterally graded multilayer focusing mirror is placed in BL29XUL of SPring-8. The engineered focused beam is approximately 7 nm at 20 keV, and optimal focusing conditions with a sub-10 nm beam size may be maintained for a minimum of half a day (Mimura *et al.*, 2010 ▸; Yamauchi *et al.*, 2011 ▸). At the SPring-8 Angstrom Compact Free-Electron Laser (SACLA), a focal spot of 5.8 nm in the unidimensional direction and 6.5 nm × 6.9 nm in two dimensions has been demonstrated to be achievable (Inoue *et al.*, 2020 ▸; Yamada *et al.*, 2024 ▸).

The recent reference indicates that MLKB mirrors may attain focal spots as small as sub-50 nm, and perhaps even below 10 nm methodologically. It is imperative to underscore that the mechanical stability and vibration avoidance strategy of the mirror system are significant, especially for the cooling nanofocusing system at HEPS ID23. In contrast to direct cooling (Lee *et al.*, 2000 ▸), indirect cooling involves the circulation of the cooling fluids within a cooling channel rather than directly on the silicon crystal or mirror (Khosroabadi *et al.*, 2024 ▸; Lee *et al.*, 2001 ▸). This method confers several advantages, such as straightforward and cost-effective fabrication, as well as a reliable metal-to-metal seal, and is extensively employed in third- or fourth-generation synchrotron sources. The cooling fluids include helium gas (Toellner *et al.*, 2006 ▸), water (Zhang *et al.*, 2013 ▸) and liquid nitro­gen (Khosroabadi *et al.*, 2024 ▸). Alongside cooling channels that connect copper blocks to the silicon structure (Liu *et al.*, 2024 ▸), copper braids (Lena *et al.*, 2021 ▸) or copper stripes (Takeuchi *et al.*, 2011 ▸) function as a thermal conduit to the cooling assembly. For the first-phase beamline stations at HEPS, smart-cut mirrors with notched substrates and water cooling are the primary choice for white beam mirrors (Wang *et al.*, 2022 ▸). Eutectic gallium-indium (eGaIn) is employed to leverage its advantages, including excellent wettability, high thermal conductivity and vibration isolation properties, by filling the notches on the mirrors (Zhang *et al.*, 2013 ▸; Zhang *et al.*, 2017 ▸). The constraints of multi-dimensional alignment and compact space, along with the stringent stability requirements and vibration isolation cooling scheme, present significant challenges in mechanical structure design for the cooling nanoscale focusing system at ID23. Therefore, this paper presents a sophisticated design of a cooling MLKB mirror system, including the mechanical structure and cooling system, which are described in Section 2. A comprehensive evaluation of the system’s performance, including mechanical performance, mirrors’ surface shape error, stability and focal spot, is presented in Section 3. A conclusion will be given in Section 4.

## 2. Structure design

**Figure 1:**
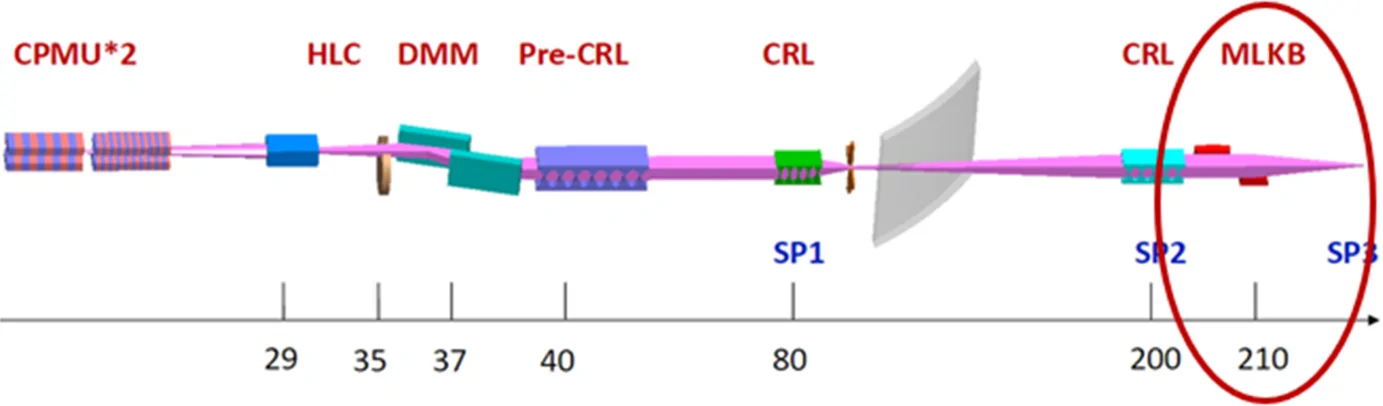
Optics layout diagram of HEPS ID23. Distances shown are in metres.

The optical layout of the nanometre mode at HEPS ID23 is shown in Fig. 1 ▸. The MLKB mirror system is located 210 m from the beam source, aiming to focus the secondary light source generated by the compound refractive lens (CRL) at 95.5 m to the nanometre scale. The MLKB mirrors consist of two orthogonal grazing incidence mirrors, a horizontal focusing KB mirror [HFM; size: 70 mm (L) × 40 mm (W) × 50 mm (H)] and a vertical focusing KB mirror [VFM; size: 120 mm (L) × 40 mm (W) × 40 mm (H)]. The mirrors are coated with W/B_4_C. The gamma ratio is 0.5, and the total number of periods is 125. The mirrors are shaped as elliptical cylinders. The lateral *d*-spacing design is graded. The *d*-spacing at the mirror centre is designed as 2.6 nm. For the VFM, the designed object distance *p* is 114.5 m and the image distance *q* is 0.175 m. For the HFM, *p* equals 114.6 m, and *q* equals 0.075 m. The grazing incidence angle is specified as 10.925 mrad. The MLKB mirror system is working at a photon energy of 21.8 keV.

### 2.1. Mechanical structure design

**Figure 2:**
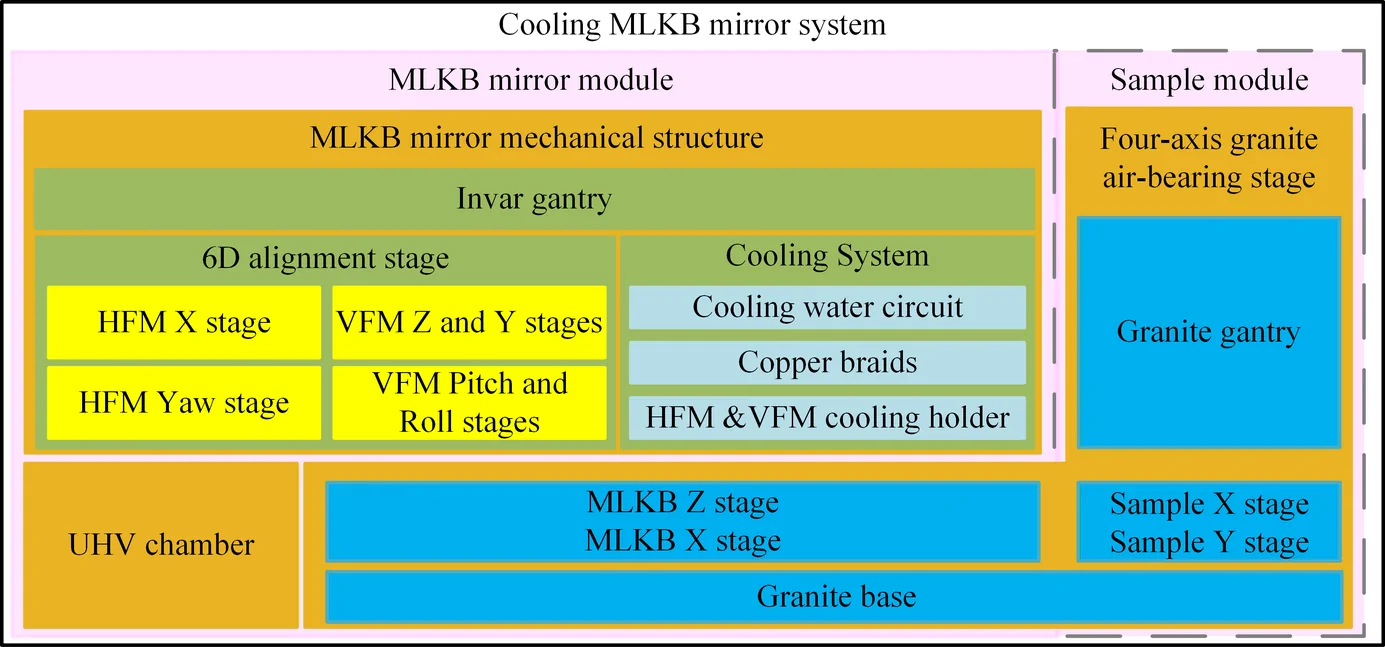
Schematic of the cooling MLKB mirror system in ID23.

Fig. 2 ▸ illustrates a schematic of the cooling MLKB mirror system. The MLKB mirror module comprises a four-axis granite air-bearing stage, a UHV chamber, and an MLKB mirror mechanical structure. Parts of the four-axis granite air-bearing stage belong to the sample module and offer a two-dimensional stage (Sample X stage and Sample Y stage) for the sample and a two-dimensional stage (MLKB Z stage and MLKB X stage) for the MLKB mirror mechanism. The MLKB mirror mechanical structure includes an Invar gantry, a six-dimensional (6D) alignment stage, and a cooling system. The 6D alignment stage is designed for the adjustment of the MLKB mirrors. The cooling system is an essential component. We shall describe these components sequentially.

**Figure 3:**
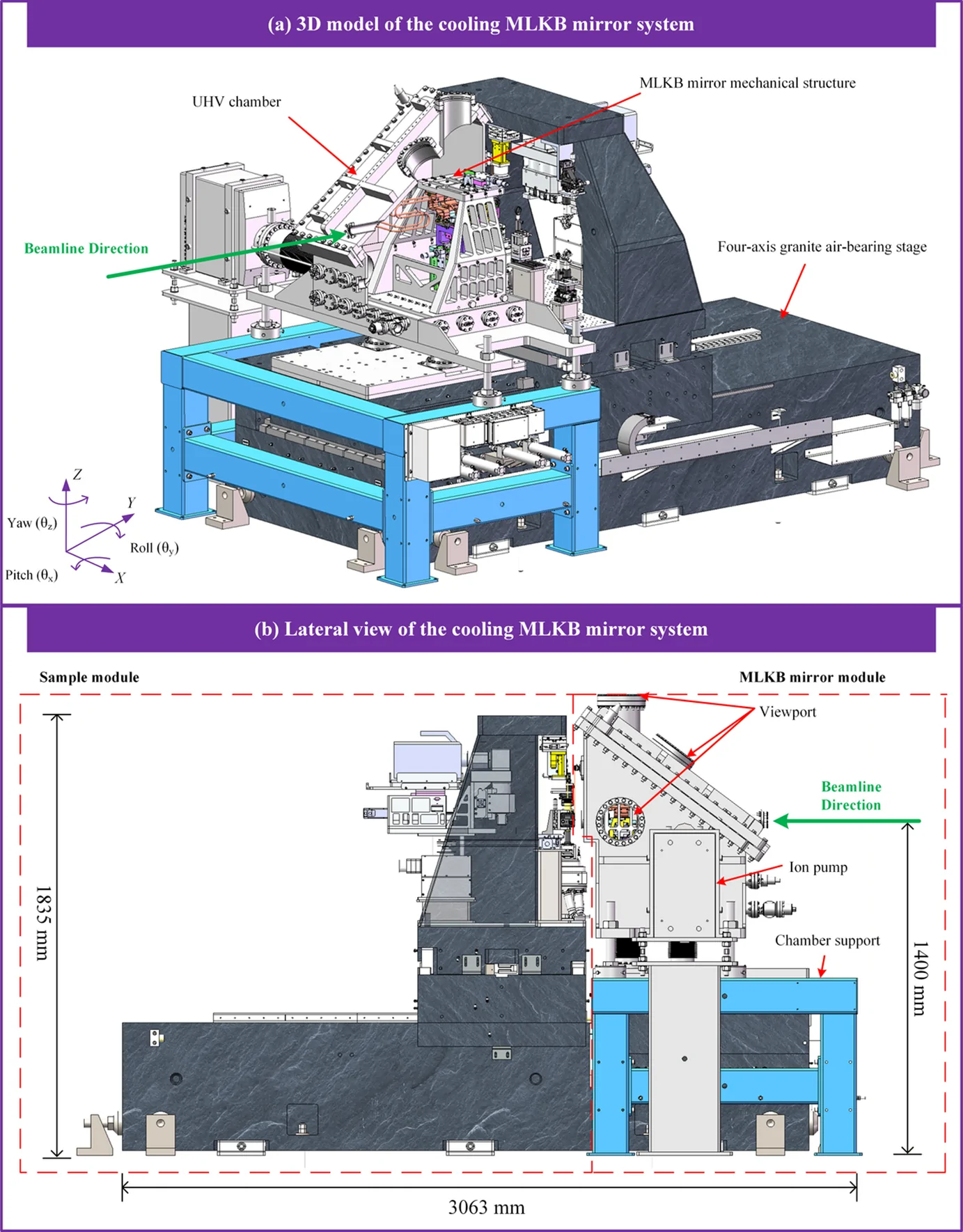
(*a*) 3D model and (*b*) lateral view of the cooling MLKB mirror system

Fig. 3 ▸ displays a comprehensive 3D model with a lateral view of the cooling MLKB mirror system. Positional correlations among the four-axis granite air-bearing stage, the UHV chamber and the MLKB mirror mechanical structure are illustrated in Fig. 3 ▸(*a*). A lateral view is provided in Fig. 3 ▸(*b*). The dimensions of length and height are labelled. The locations of the viewport, ion pump and chamber base are also shown.

**Figure 4:**
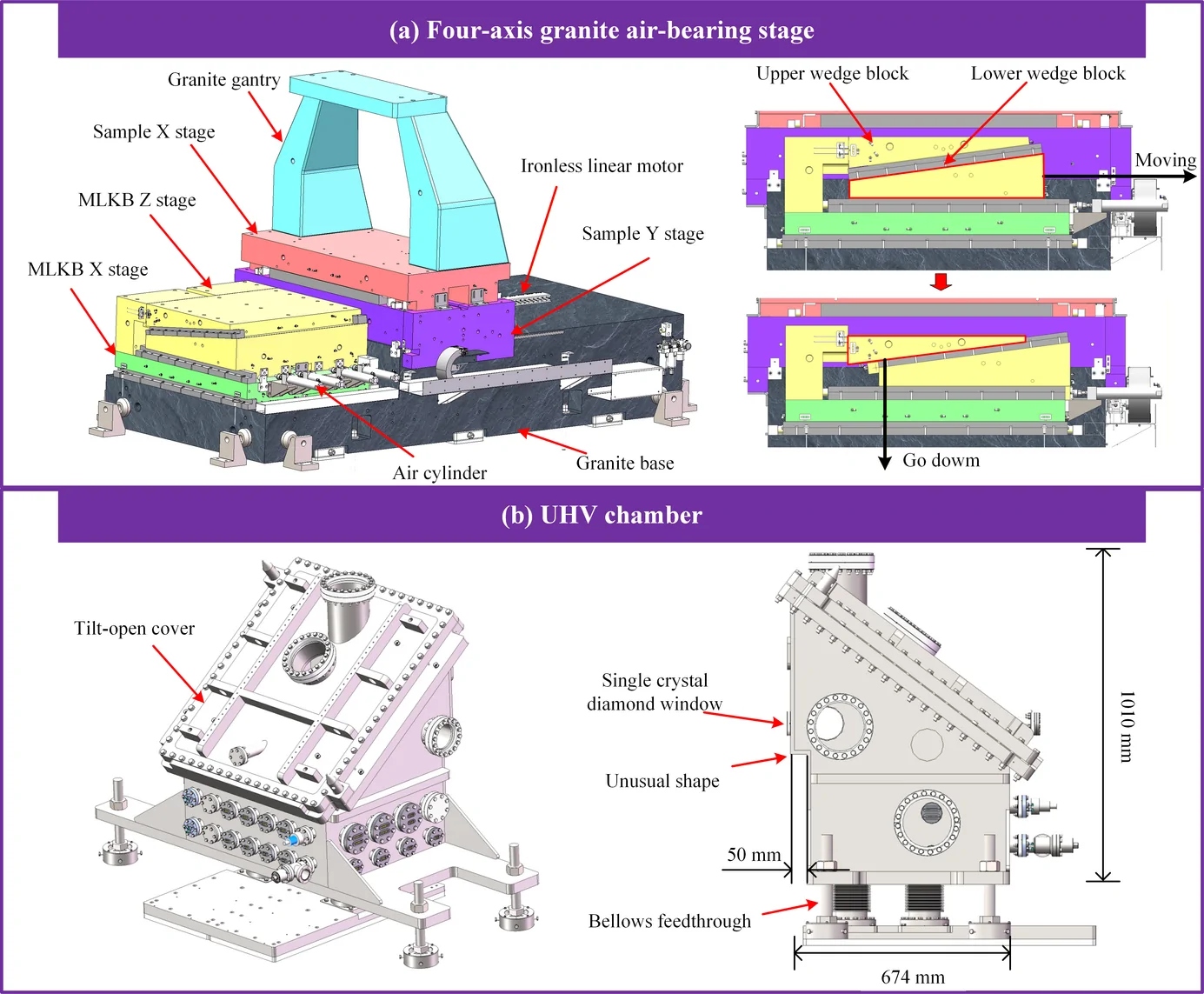
3D models of the (*a*) four-axis granite air-bearing stage and (*b*) UHV chamber.

Fig. 4 ▸ shows a 3D model of the four-axis granite air-bearing stage and the UHV chamber. In Fig. 4 ▸(*a*), several components of the air-bearing stage are distinguished by various colours. The granite base is identical for the both sample module and MLKB mirrors module, with granite platforms above. Ironless linear motors (AUS, from the Akribis company) are applied in every axis. The MLKB Z stage differs slightly from other axis stages. Air cylinders contribute to the push force to prevent sliding down. A motor drives the lower wedge block instead of the upper one to ensure that the *X*-axis position remains unaffected by *Z*-axis movement. A granite gantry is constructed above the sample alignment stage to provide an installation space for commercial translation stages for different experiments, as seen in Fig. 2 ▸. In Fig. 4 ▸(*b*), some parameters of the abnormal shape of the UHV chamber are provided. In addition to conventional considerations, the UHV chamber features a tilt-open cover and an unusual form downstream. The tilt-open cover design enhances assembly and alignment efficiency. A 50 mm recessed, unusual-shaped design facilitates sample placement. The 200 µm-thick single crystal diamond window has a clear diameter of 10 mm for the passage of the beam. The UHV chamber is decoupled from the MLKB mirror’s mechanism using bellows feedthroughs to prevent the chamber vibrations from compromising the stability of the internal mechanism (Tang *et al.*, 2023 ▸).

**Figure 5:**
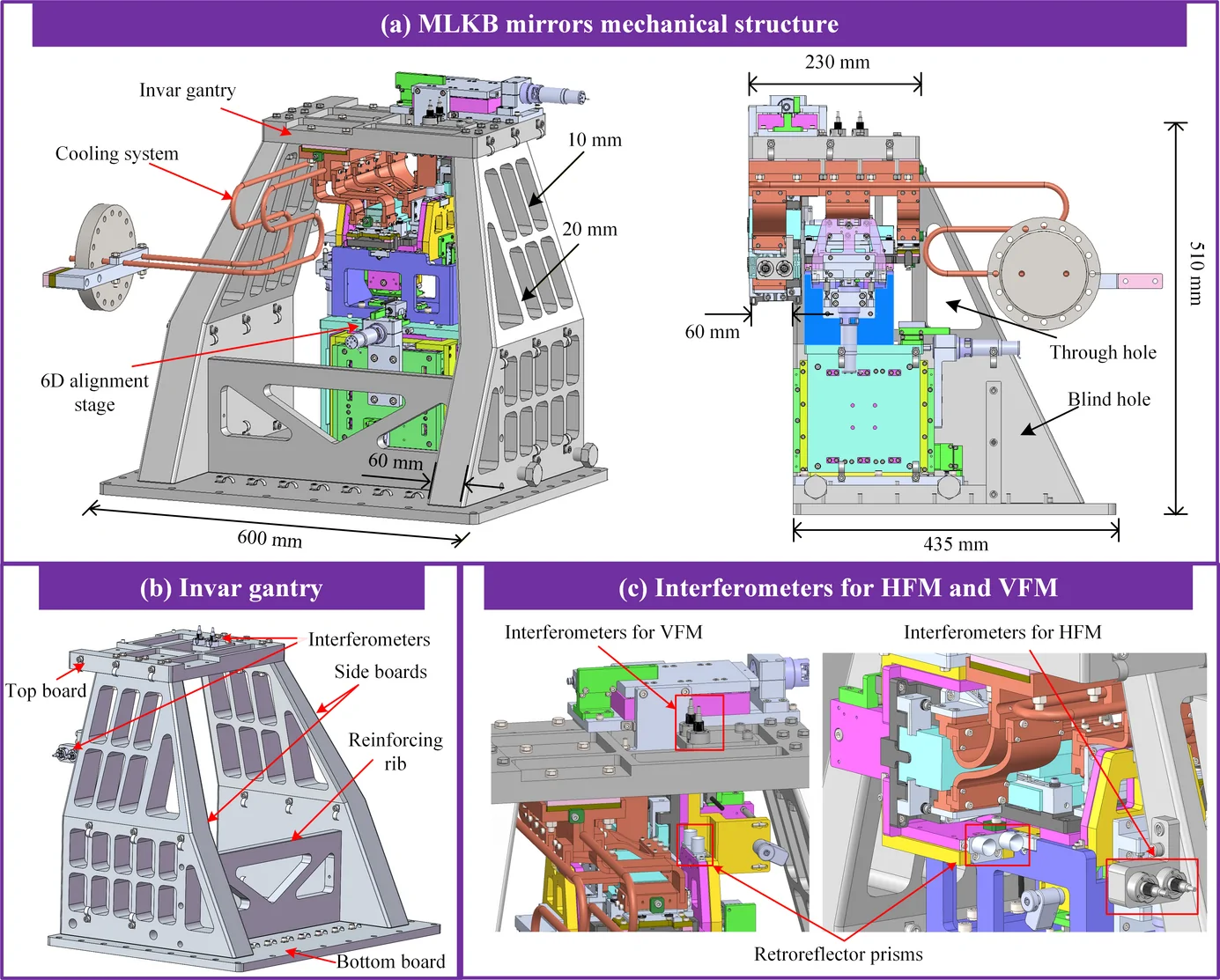
3D models of the (*a*) integrated MLKB mirrors mechanical structure, (*b*) Invar gantry and (*c*) interferometers for HFM and VFM.

**Figure 6:**
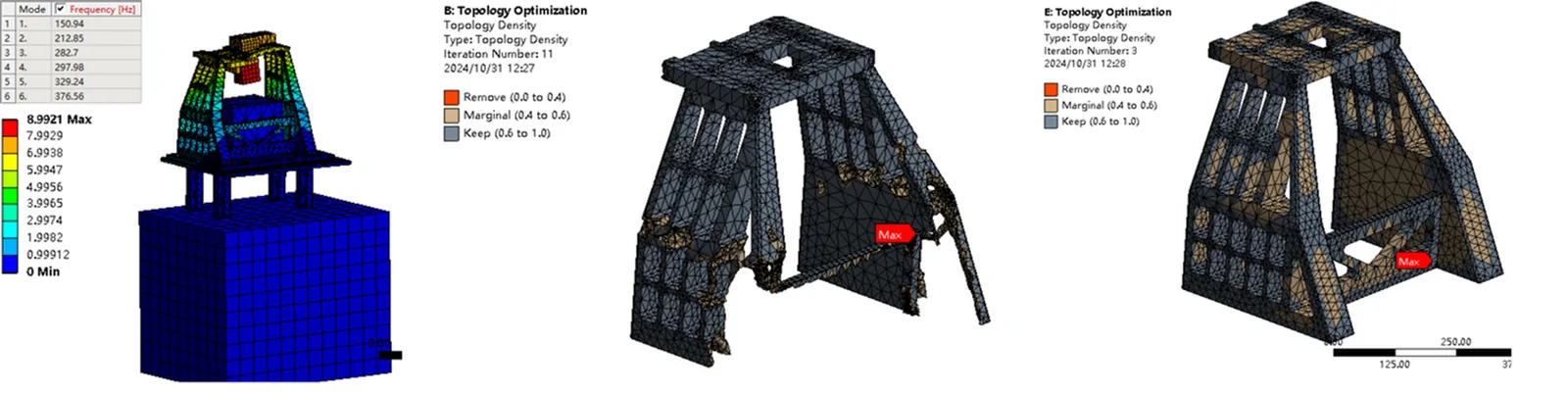
(*a*) Modal FEA, (*b*) mass-targeted topology optimization and (*c*) modal-targeted topology optimization of the Invar gantry frame in FEA.

Fig. 5 ▸ shows the 3D model of the integrated MLKB mirror mechanical structure and size parameters of the Invar gantry. The Invar gantry is divided into five parts to process: a top board, two side boards, a bottom board, and a reinforcing rib. The *X* stage and yaw stage for the HFM are mounted inversely on the Invar gantry. The size parameters of the Invar gantry are provided in Fig. 5 ▸(*a*). In Fig. 5 ▸(*c*), two pairs of interferometer sensor heads and the cooling system are affixed to it. Consequently, stability and lightweight construction of the Invar gantry is paramount. Through iteration and topology optimization in finite-element analysis (FEA), the structure in modal-targeted topology optimization lacks the red removable material shown in Fig. 6 ▸. The thickness of the ribs on the side boards is either 10 mm or 20 mm. The holes on the upper part of the side boards are through holes, while the lower part has blind holes with a thickness of 15 mm. The modal frequency exceeds 150 Hz, while the mass is only 148 kg.

**Figure 7:**
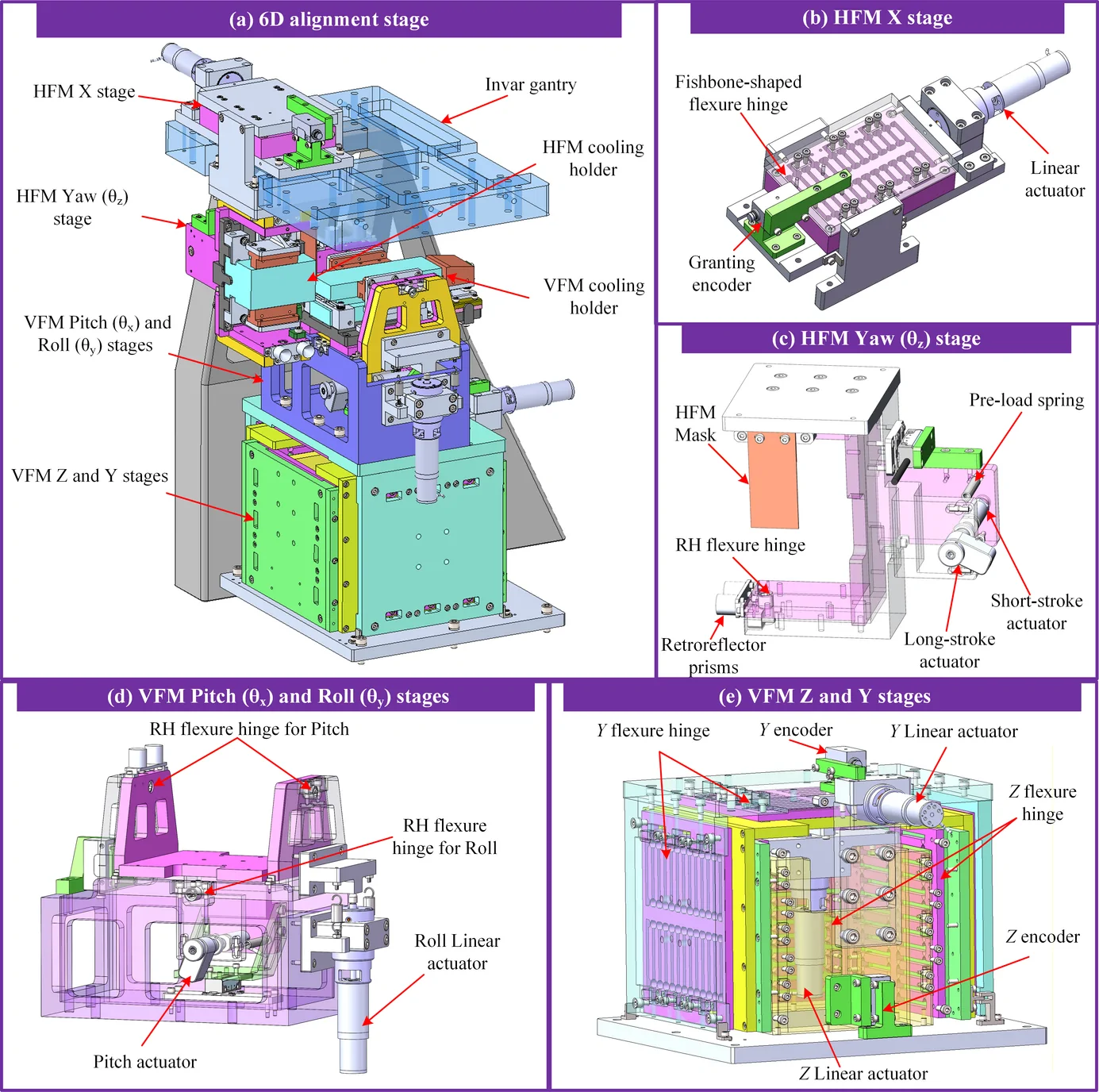
3D model of the (*a*) 6D alignment stage, (*b*) HFM X stage, (*c*) HFM yaw stage, (*d*) VFM pitch and roll stages and (*e*) VFM Z and Y stages.

Fig. 7 ▸ shows a 3D model of the integrated 6D alignment stage and all its components. The six degrees of freedom adjustment of the MLKB mirrors is made feasible by the 6D alignment stage, which consists of an X stage and a yaw stage for HFM, pitch and roll stages, and Z and Y stages for VFM. Linear actuators (Irelec AC300) operate fishbone-shaped flexure hinges (Shu *et al.*, 2019 ▸) to provide limited movement in the translation stage. The rotation stages use the precision flexure pivot bearing manufactured by the Riverhawk company (cantilevered flexures 5012-400) as rotational bearings. The springs in Fig. 7 ▸(*c*) are used to give a pre-load for the VFM pitch stage and HFM yaw stage. In principle, throughout the motor’s thrust range, a spring should possess maximal stiffness to guarantee the mechanism’s overall stability. For adjustment of the grazing incidence angle of the two mirrors, a long-stroke actuator (Newport 8301-UHV-KAP picomotor actuator) and a short-stroke but high-accuracy actuator (PI-841) drive the mirrors together. All six dimensions have encoders to monitor the position. A mask with a thickness of 1 mm is incorporated in the HFM yaw stage to protect the HFM.

### 2.2. Cooling system

**Figure 8:**
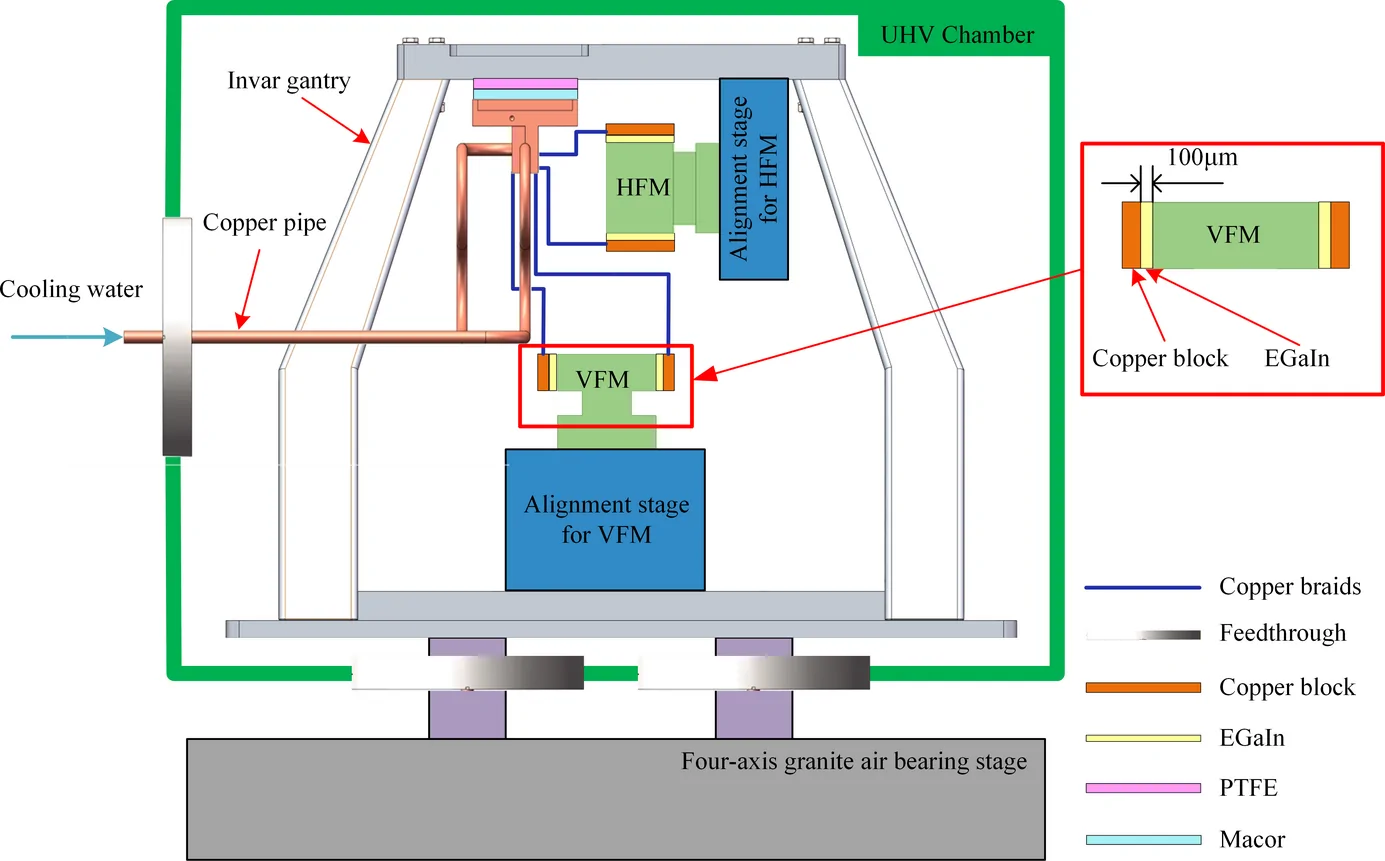
Schematic diagram of the cooling system.

Fig. 8 ▸ shows a schematic diagram of the cooling system. The cooling pathway sequentially traverses the VFM/HFM, eGaIn, copper block, copper braids and cooling water circuit. Two movable mechanisms exist: an alignment stage for HFM/VFM inside the UHV chamber and a four-axis granite air-bearing stage external to the chamber. Three strategies are used for motion decoupling and vibration isolation. First, the eGaIn occupies the space between the mirror and the copper block, which has a thickness of about 100 µm (Zhang *et al.*, 2016 ▸). The eGaIn exhibits great thermal conductivity, excellent wettability and particularly lacks clamping force; hence, the copper block is engaged indirectly with mirrors using the eGaIn rather than being directly connected. Secondly, in order to decouple the sophisticated motion of the 6D alignment stage within the UHV chamber, a 2 mm thickness of copper braids is used to link the cooling copper pipe and the copper block. To enhance the separation of motion from the granite air-bearing stage external to the chamber and to facilitate the compact spatial configuration of the mirrors, sample and chamber, the cooling water circuit is designed as a copper pipe with a multi-bend structure that goes inside the vacuum. One end is installed on the Invar gantry inversely, while the other end is welded with a flange fixed to the UHV chamber.

**Figure 9:**
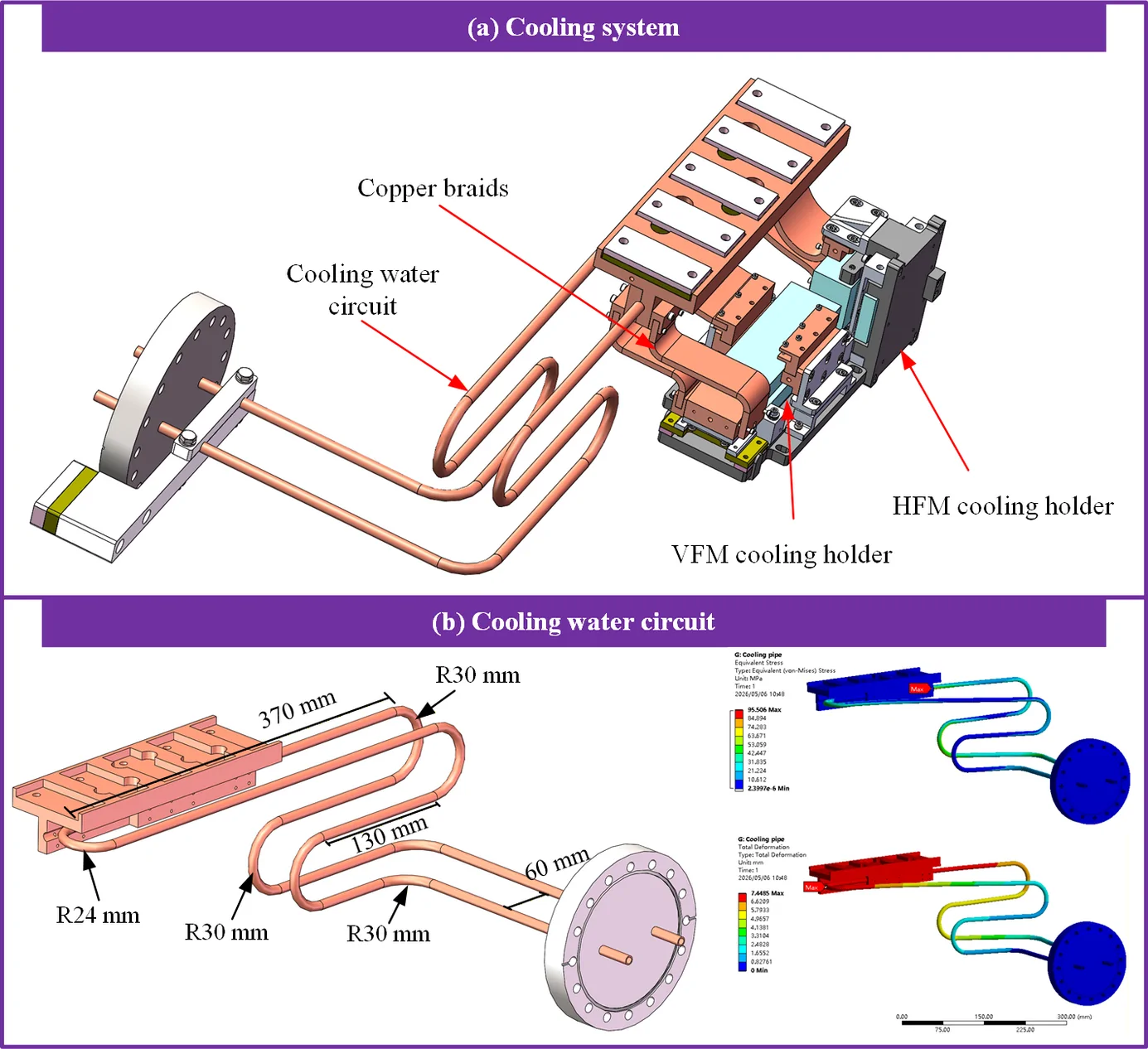
(*a*) 3D model of the cooling system. (*b*) 3D model and FEA of the cooling water circuit.

**Figure 10:**
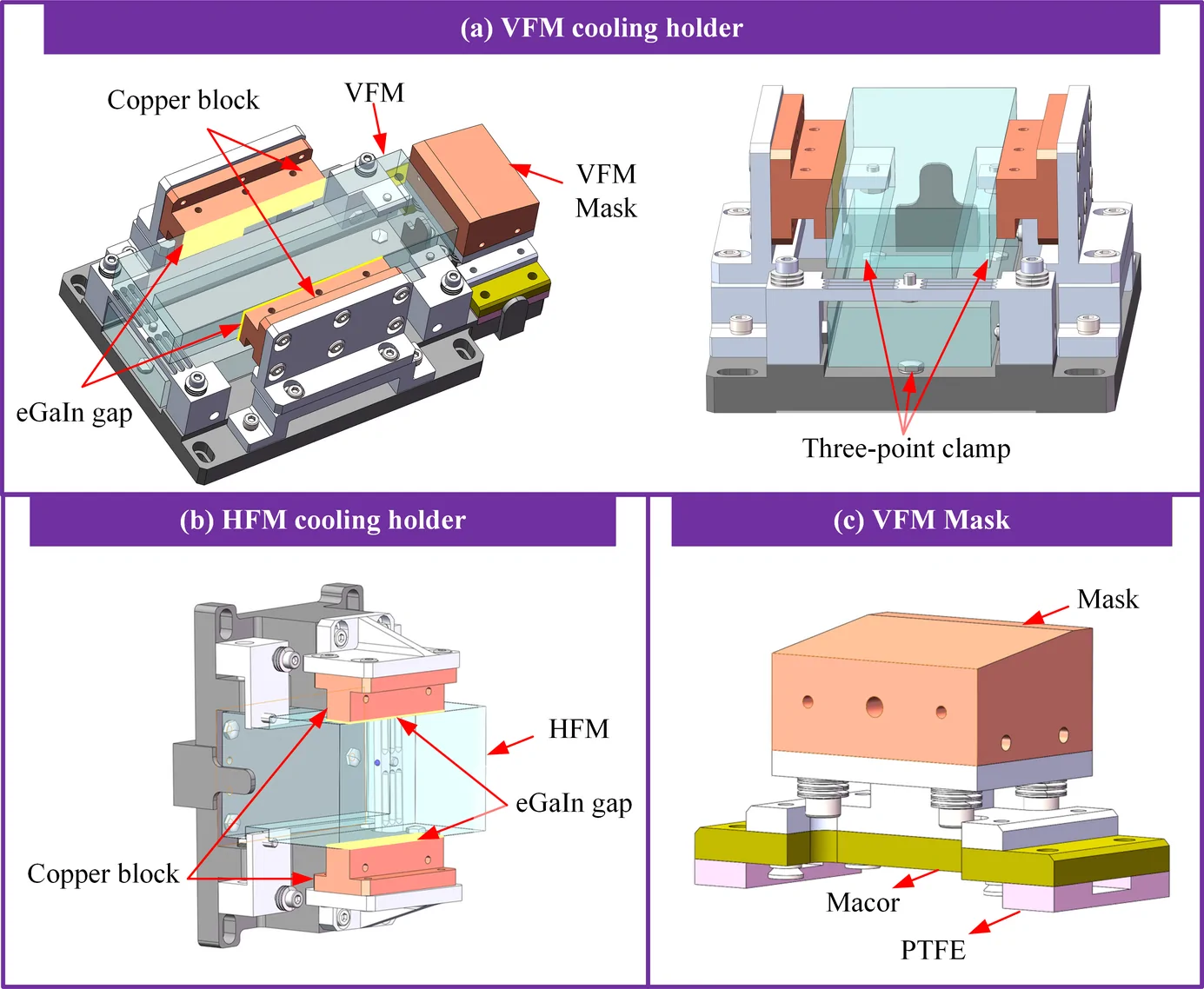
3D model of the (*a*) VFM cooling holder, (*b*) HFM cooling holder and (*c*) VFM mask.

A 3D model of the cooling system, comprehensive size parameters and statistical FEA simulation of the cooling copper circuit featuring a multi-bend copper pipe are illustrated in Fig. 9 ▸. The pipe has an outer diameter of 8 mm and an inner diameter of 6 mm. The total length of the cooling copper pipe is about 2250 mm. FEA of the copper pipe stress demonstrates its compliance with the movement range requirement. The cooling water operates at a flow rate of 4.5 L min^−1^ with a temperature of 25°C. To mitigate the vibration and thermal conduction, the cooling water circuit is indirectly connected to the Invar gantry frame through a Polytetra­fluoro­ethyl­ene isolation material and a Macor ceramic insulation material. 3D models of the HFM/VFM cooling holder are also displayed in Fig. 10 ▸. The dimensions of the copper block are calculated and simulated conditionally (Wang *et al.*, 2022 ▸). The eGaIn lacks a force clamp, resulting in mirror surface deformation only from the three-point clamp during installation. A mask is mounted in front of the VFM and also isolated by Macor and PTFE. A typical periodic pulse loading mode of the thermal load chopper in HEPS ID23 is 20 ms/80 ms. The thermal deformation of the HFM and VFM surfaces was simulated under this condition, as detailed by Liao *et al.* (2024 ▸). The simulated peak-to-valley (PV) values of the mirror surface shape error are 0.11 nm for the HFM and 0.60 nm for the VFM.

## 3. System performance

### 3.1. Mechanical performance

**Figure 11:**
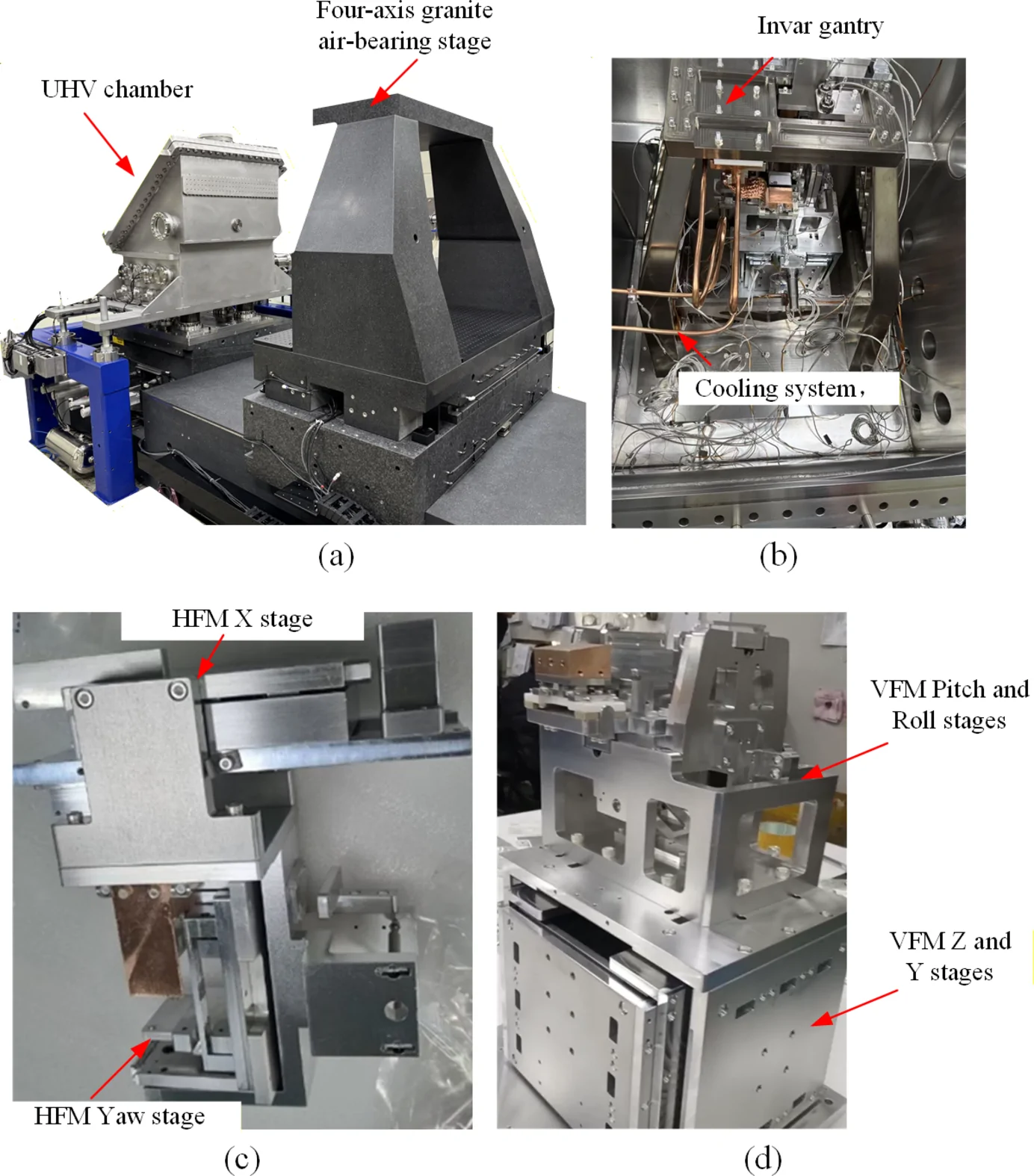
Photographs of the (*a*) MLKB mirror system, (*b*) MLKB mirror mechanism, (*c*) HFM 2D alignment stage and (*d*) VFM 4D alignment stage.

The entire system has been installed and collimation has been executed as shown in Figs. 11 ▸ and 12 ▸. The mechanism performance, encompassing the movement range, repeatability and resolution of the 6D alignment stage and four-axis granite air-bearing stage, is presented in Table 1 ▸. The positional repeatability surpasses 2 µm, and the angular repeatability exceeds 2 µrad, excluding the VFM roll stage. The positional resolution exceeds 1 µm, and the angular resolution surpasses 1 µrad. It is important to note that the resolution of the encoder system on each axis is 1 nm. The resolution tests are conducted when the increment is 1 µm or 1 µrad, and the distinction step is evident. The ultimate resolution of the mechanism system may exceed these, but we did not conduct testing.

### 3.2. Surface shape error of mirrors induced by clamping

**Figure 12:**
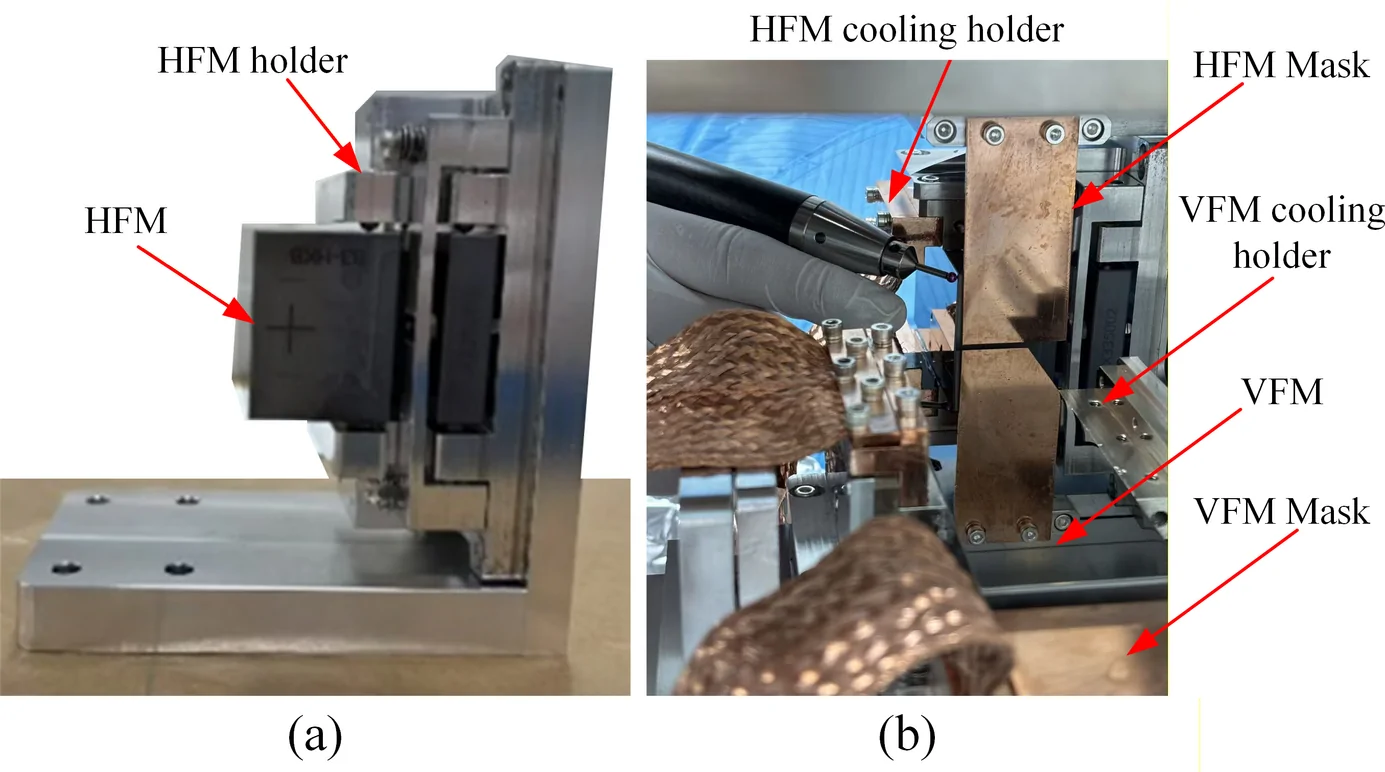
(*a*) HFM holder. (*b*) VFM or HFM cooling holder.

**Figure 13:**
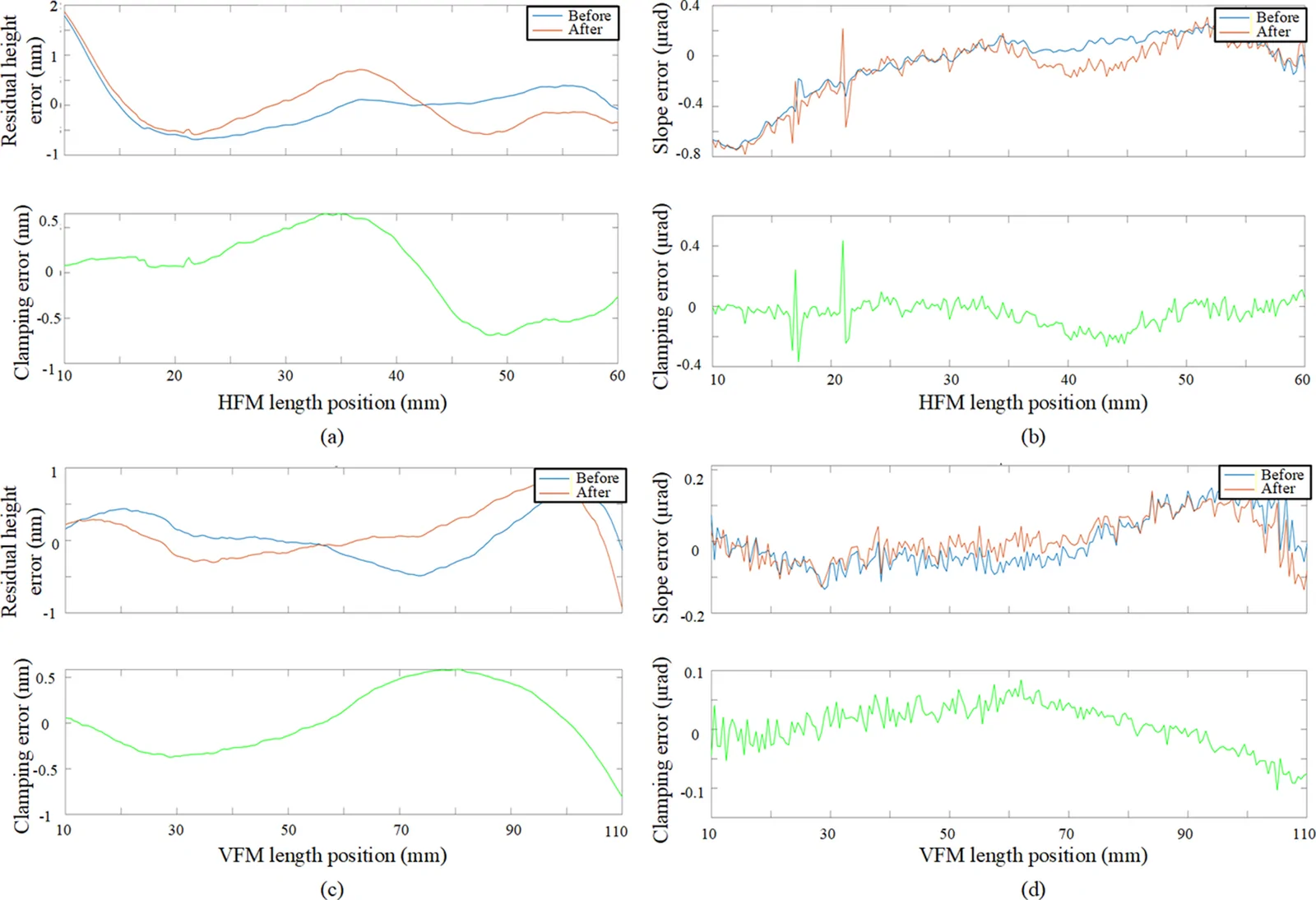
(*a*) HFM height error, (*b*) HFM slope error, (*c*) VFM height error and (*d*) VFM slope error.

The cooling holders are illustrated in Fig. 12 ▸. A long trace profiler (LTP) (Yang *et al.*, 2014 ▸; Yang *et al.*, 2016 ▸) is used to measure the surface shape of the mirrors, with findings illustrated in Fig. 13 ▸. Figs. 13 ▸(*a*)–13(*d*) present the HFM height error, HFM slope error, VFM height error and VFM slope error. In each figure, the upper diagrams compare residual height error or slope error before and after clamping, whereas the lower diagrams illustrate the surface shape defects induced by clamping. The RMS and PV values of the surface shape error are summarized in Table 2 ▸. ‘Error before clamping’ in Table 2 ▸ denotes the mirror surface error before clamping, corresponding to the results indicated by the blue lines in Fig. 13 ▸. The ‘clamping-induced error’ represents the disparity between the values before and after the clamping process, corresponding to the results of the green line in Fig. 13 ▸. The RMS and PV values of the mirror surface error post-clamping, as indicated by the red lines in Fig. 13 ▸, cannot be simply aggregated by these two parts; but the red line is the total of the blue line and the green line. The RMS and PV values of the actual post-clamping mirror surface error are potentially superior or worse than the error before clamping, but will not exceed the outcomes of direct addition. The mirror surface shape is highly susceptible to external forces; varying the clamping will change the surface shape. Nevertheless, the mirrors exhibit a well polished surface finish. The clamping structure introduces an RMS height error of less than 0.5 nm and a height PV of less than 1.5 nm. The clamping-induced RMS slope error is below 0.1 µrad, while the PV remains under 1 µrad.

### 3.3. Stability

**Figure 14:**
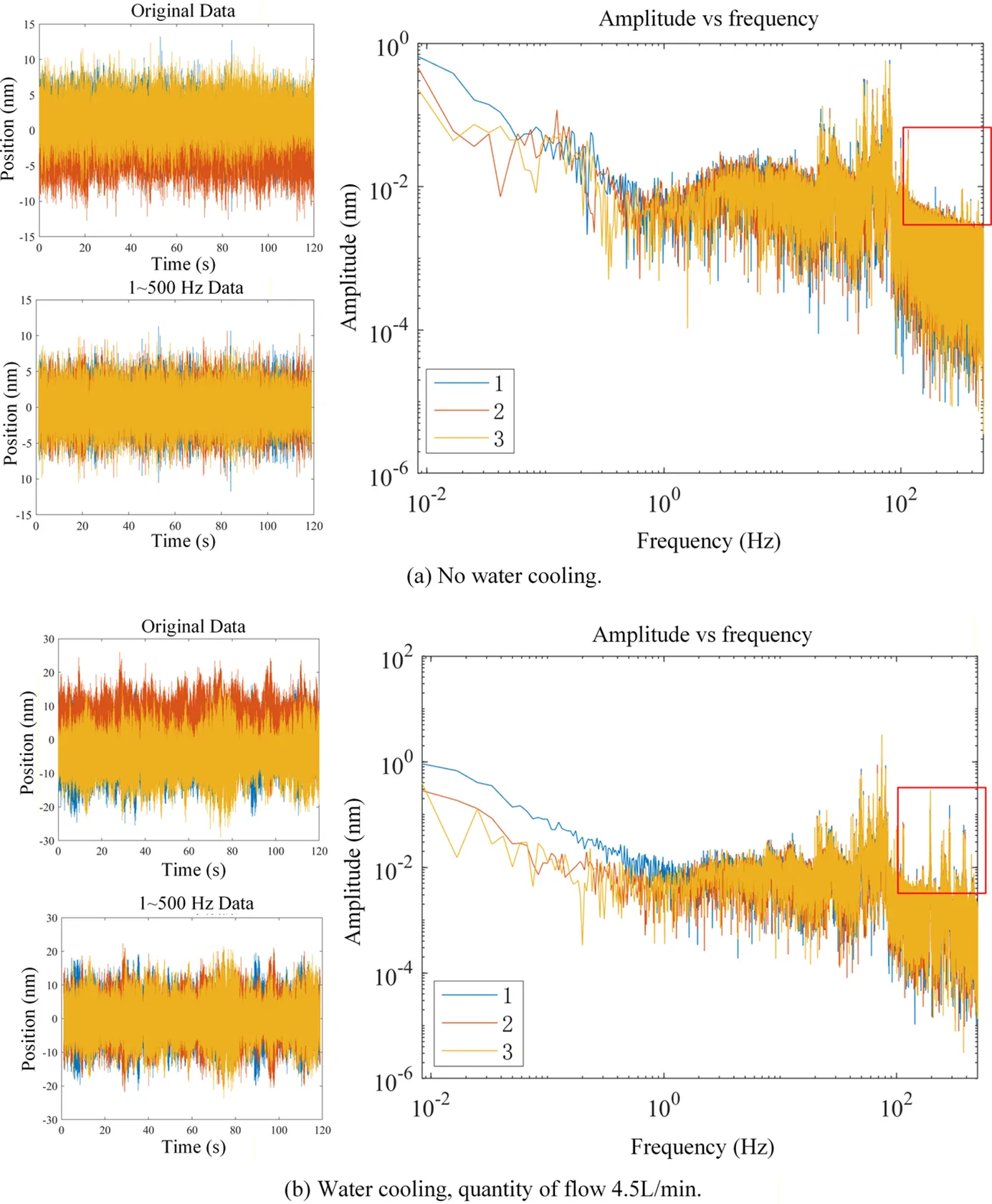
The 2 min stability assessments of the HFM. (*a*) Stability of position without water cooling. (*b*) Stability of position with a water cooling rate of 4.5 L min^−1^.

Stability is evaluated using Attocube interferometers through a sequence of tests. The performance of the inverted installed HFM mechanism on the Invar gantry frame indicates the system’s stability, as the results encompass the impacts of both the cooling system and the stability design. Fig. 14 ▸ illustrates results of the stability tests of the HFM conducted during a brief duration of 2 min with a water cooling rate of 4.5 L min^−1^ or without water cooling. Each image in Figs. 14 ▸(*a*) and 14(*b*) consist of three components: the original data are displayed in the top-left corner; the lower-left part depicts the stability data ranging from 1 Hz to 500 Hz of the original data; and the central part illustrates the amplitude versus frequency curves. Each set comprises three measurements, which are represented by different colours. The analysis indicates that the cooling water flux predominantly induces vibrations in the high-frequency band, exceeding 100 Hz. The quantitative measurements of the vibration of the HFM with water cooling and without water cooling are 2.40 nm and 5.37 nm from 1 Hz to 500 Hz, respectively. Additionally, tests of the 2 min positional vibration of the VFM, both with or without water cooling, as well as the 1 h positional and angular vibration of the system (HFM) under the same conditions, have been conducted, and the results are consolidated in Table 3 ▸.

A second comparison that should be highlighted is that in 1 h tests the positional and angular stability of the system without water cooling measured 16.26 nm and 683.10 nrad from 0 Hz to 500 Hz, while these values reduced to 3.08 nm and 25.18 nrad with a 1 Hz to 500 Hz filter. The pre-filter data suggest gradual drifts, usually falling within the 0 Hz to 1 Hz range. The data ranging from 1 Hz to 500 Hz can indicate the system’s stability (Bueno *et al.*, 2021 ▸; Geraldes *et al.*, 2021 ▸; Saveri Silva *et al.*, 2021 ▸; Kristiansen *et al.*, 2015 ▸). Significant pre-filter instability and slow drift are observed due to ambient temperature variations in the absence of cooling. Conversely, with water cooling, the positional and angular stability were recorded at 12.70 nm and 129.41 nrad from 0 Hz to 500 Hz, respectively, and reduced to 5.96 nm and 86.76 nrad following a 1–500 Hz high-pass filter. Water cooling reduces the slow drift due to enhanced temperature regulation, and it simultaneously increases system instability from water flow vibrations.

The final comparison to highlight is that the short-term positional stability of the VFM without and with water cooling can achieve 0.78 nm and 1.23 nm, respectively, from 1 Hz to 500 Hz; the measurements of the HFM under the same conditions are 2.40 nm and 5.37 nm. The stability of the VFM is better than that of the HFM. The different results between the VFM and HFM demonstrate the stability of the engineered Invar gantry. The short-term stability results may hold greater significance, as the sample scanning duration for considerable experiments is only a few minutes.

**Figure 15:**
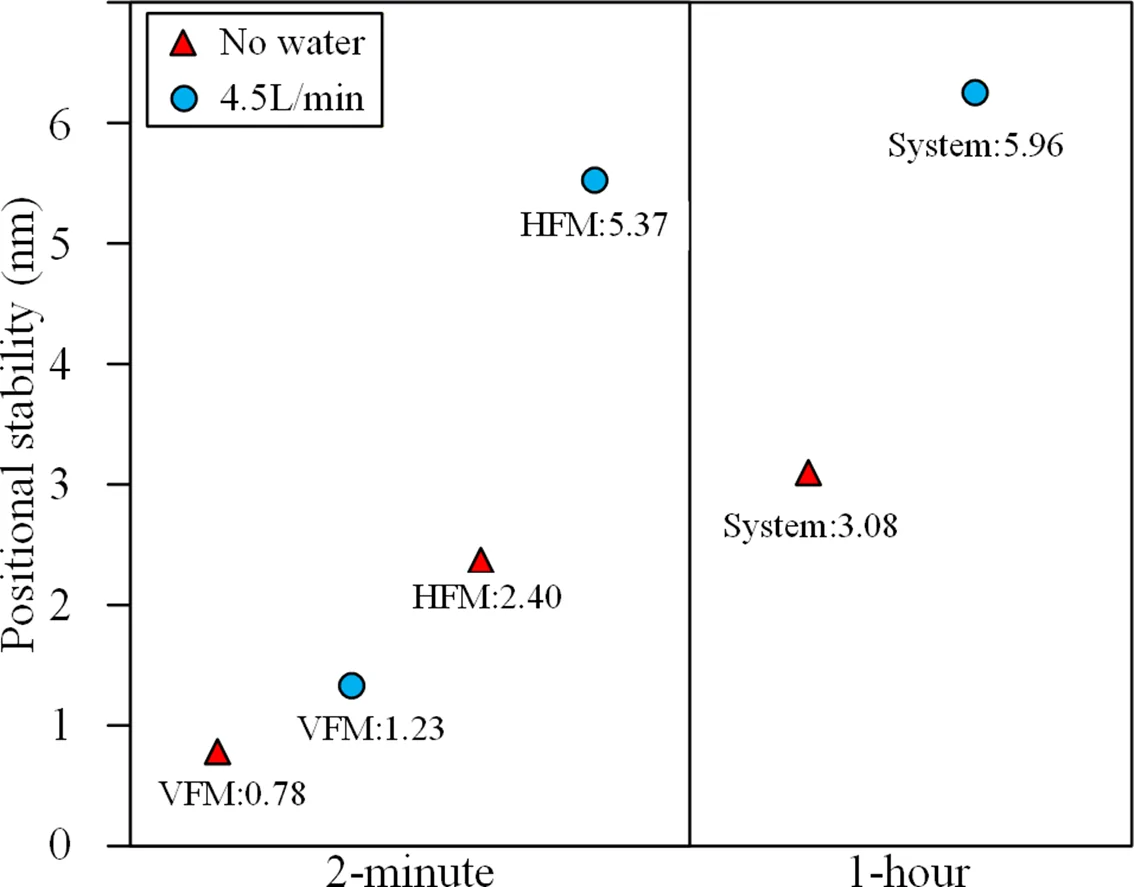
Positional stability from 1 Hz to 500 Hz under varying water flow circumstances and different testing durations.

Fig. 15 ▸ provides a more intuitive presentation of the positional stability ranging from 1 Hz to 500 Hz under different water flux conditions. The difference between the HFM and VFM results for the 2 min tests illustrates the stability of the inverse installation structure configuration within the gantry frame. The distribution patterns of the red triangular points and the blue circular points quantitatively reveal the impact of water flow on the structure. Moreover, the 1 h tests of the VFM are deficient due to the interferometers within the UHV chamber losing the reflected laser light during roll angle alignment. Nevertheless, the VFM 1 h tests are unnecessary as the interferometers for the HFM are accessible. The stability of the HFM is inferior to that of the VFM; thus, utilizing the HFM interferometer as a reference is sufficient.

### 3.4. Spot size

**Figure 16:**
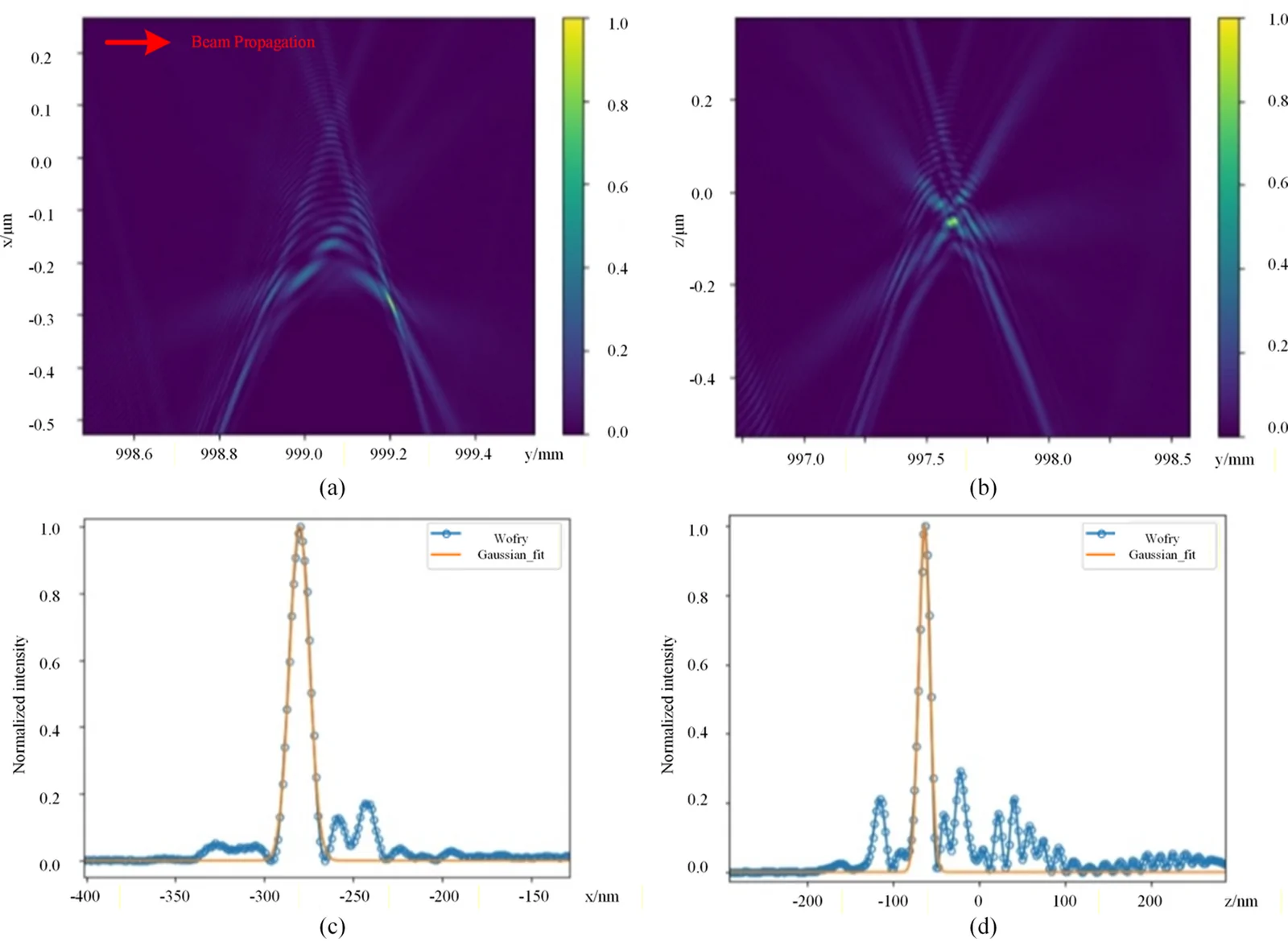
The intensity distribution near the focus of the (*a*) HFM and (*b*) VFM, and intensity profiles at the focus of (*c*) the HFM and (*d*) the VFM.

Finally, speckle scanning metrology (Wang *et al.*, 2015 ▸) is utilized to determine the focal spot size. The size of the focal spot has been subjected to multiple tests, and Fig. 16 ▸ illustrates one of the results. The intensity distribution near the focus of the HFM and VFM is illustrated in Figs. 16 ▸(*a*) and 16(*b*). The intensity profiles at the focus of the HFM and VFM are shown in Figs. 16 ▸(*c*) and 16(*d*). The focal spot size reaches 13.39 nm × 15.15 nm (H × V) at a photon energy of 21.8 keV. The vertical and horizontal focus positions exhibit an offset of approximately 2 mm, derived from speckle scanning metrology. The sub-20 nm focal spot further proves the performance of the cooling MLKB mirror system.

## 4. Conclusion

This paper presents the structural design of a cooling MLKB mirror system. The system is characterized by two primary features for enhanced stability: a high-stability structure design and a cooling system employing eGaIn, copper braids and a cooling water circuit with a multi-bend copper pipe. The cooling holder and 6D alignment stage were installed successfully. The system has the required mechanical capabilities. The mirror holders introduce less than 0.5 nm RMS and 1.5 nm PV in height error, and less than 0.1 µrad and 1 µrad PV in slope error. A series of stability tests were applied to verify the mechanism structure. The disparity between the VFM (0.78 nm without water cooling and 1.23 nm with water cooling) and HFM (2.40 nm without water cooling and 5.37 nm with water cooling) throughout 2 min tests illustrates the stability of the inverse structure on the Invar gantry. The 1 h stability test of the HFM (3.08 nm without water cooling and 5.96 nm with water cooling) quantitatively reveals the impact of water flow on the structure. Consequently, the system achieved positional and angular stability of 5.96 nm (RMS) and 86.76 nrad (RMS) from 1 Hz to 500 Hz, respectively, during a 1 h test under a water-cooling flux of 4.5 L min^−1^. Conclusively, the mechanism performance was validated by achieving a focal spot size of 13.39 nm × 15.15 nm at a photon energy of 21.8 keV.

## Figures and Tables

**Table 1:** Mechanism performance of the 6D alignment stage and four-axis granite air-bearing stage

Stage	Movement Range	Repeatability	Resolution
VFM Z	−0.8 mm to 2 mm	0.021 µm	≤1 µm
VFM Y	−3 mm to 0.5 mm	0.701 µm	≤1 µm
VFM pitch	±10 µrad	0.231 µrad	≤1 µrad
VFM roll	±20 µrad	6.452 µrad	≤1 µrad
HFM X	±0.5 mm	0.014 µm	≤1 µm
HFM Y	±10 µrad	1.665 µrad	≤1 µrad
MLKB X	−1 mm to 5 mm	1.1 µm	≤1 µm
MLKB Z	−3 mm to 10 mm	2.0 µm	≤1 µm
Sample X	±5 mm	2.0 µm	≤1 µm
Sample Y	0 to 1000 mm	1.2 µm	≤1 µm

**Table 2:** HFM and VFM surface shape error measured by LTP

	HFM		VFM	
	RMS	PV	RMS	PV
Residual height error before clamping (nm)	0.47	2.48	0.33	1.21
Clamping-induced height error (nm)	0.43	1.34	0.35	1.40
Slope error before clamping (µrad)	0.27	0.99	0.07	0.34
Clamping-induced slope error (µrad)	0.10	0.80	0.04	0.19

**Table 3:** Results of stability assessment

		Water cooling (0 Hz to 500 Hz)	Water cooling (1 Hz to 500 Hz)	No water cooling (0 Hz to 500 Hz)	No water cooling (1 Hz to 500 Hz)
2 min	HFM position	6.66 nm	5.37 nm	3.07 nm	2.40 nm
	VFM position	4.33 nm	1.23 nm	2.43 nm	0.78 nm
1 h	System position	12.70 nm	5.96 nm	16.26 nm	3.08 nm
	System angle	129.41 nrad	86.76 nrad	683.10 nrad	25.18 nrad
